# *Candida albicans* Delays HIV-1 Replication in Macrophages

**DOI:** 10.1371/journal.pone.0072814

**Published:** 2013-08-23

**Authors:** Christian Rodriguez Rodrigues, Federico Remes Lenicov, Carolina Jancic, Juan Sabatté, Mercedes Cabrini, Ana Ceballos, Antonela Merlotti, Heidi Gonzalez, Matías Ostrowski, Jorge Geffner

**Affiliations:** 1 Instituto de Investigaciones Médicas en Retrovirus y SIDA (INBIRS), Facultad de Medicina, Universidad de Buenos Aires, Ciudad de Buenos Aires, Argentina; 2 Instituto de Inmunología, Genética y Metabolismo (INIGEM), Hospital de Clínicas “José de San Martín”, Universidad de Buenos Aires, Ciudad de Buenos Aires, Argentina; Salute San Raffaele University School of Medicine, Italy

## Abstract

Macrophages are one of the most important HIV-1 target cells. Unlike CD4^+^ T cells, macrophages are resistant to the cytophatic effect of HIV-1. They are able to produce and harbor the virus for long periods acting as a viral reservoir. Candida albicans (CA) is a commensal fungus that colonizes the portals of HIV-1 entry, such as the vagina and the rectum, and becomes an aggressive pathogen in AIDS patients. In this study, we analyzed the ability of CA to modulate the course of HIV-1 infection in human monocyte-derived macrophages. We found that CA abrogated HIV-1 replication in macrophages when it was evaluated 7 days after virus inoculation. A similar inhibitory effect was observed in monocyte-derived dendritic cells. The analysis of the mechanisms responsible for the inhibition of HIV-1 production in macrophages revealed that CA efficiently sequesters HIV-1 particles avoiding its infectivity. Moreover, by acting on macrophages themselves, CA diminishes their permissibility to HIV-1 infection by reducing the expression of CD4, enhancing the production of the CCR5-interacting chemokines CCL3/MIP-1α, CCL4/MIP-1β, and CCL5/RANTES, and stimulating the production of interferon-α and the restriction factors APOBEC3G, APOBEC3F, and tetherin. Interestingly, abrogation of HIV-1 replication was overcome when the infection of macrophages was evaluated 2-3 weeks after virus inoculation. However, this reactivation of HIV-1 infection could be silenced by CA when added periodically to HIV-1-challenged macrophages. The induction of a silent HIV-1 infection in macrophages at the periphery, where cells are continuously confronted with CA, might help HIV-1 to evade the immune response and to promote resistance to antiretroviral therapy.

## Introduction

HIV-1 infections are mainly acquired through sexual contact. After deposition of HIV-1 on the recipient mucosa, the infectious virus must cross the mucosal epithelium and interact with resident CD4^+^ T lymphocytes, macrophages and dendritic cells (DCs), the three major targets of HIV-1 infection. These cells express the receptor CD4 and the coreceptors CXCR4 or CCR5 required for HIV-1 infection [1,2]. Unlike CD4^+^ T lymphocytes, macrophages are more resistant to the cytopathic effects of HIV-1. They are able to produce and accumulate replication competent virus for long periods even in patients on highly active antiretroviral therapy (HAART) [3–5]. This feature, together with their ubiquitous distribution in all tissues, including the central nervous system, explains why macrophages play an important role in the spreading of HIV-1 infection [3–5].

Due to the limited accessibility and inefficient recovery of tissue macrophages, most studies focused on the analysis of the infection of mononuclear phagocytes by HIV-1 used macrophages differentiated from blood monocytes. The kinetics of HIV-1 replication appears to be similar in these macrophages compared with tissue macrophages [4,6]. The production of virus particles using R5 tropic virus increases linearly over time reaching a maximum at 14 days after virus inoculation. Then, macrophages sustain high levels of viral production for at least 2 months post-infection [7]. Infected macrophages are not only able to produce infective viruses. Macrophages together with resting (naive and memory) CD4^+^ T cells constitute latent HIV-1 reservoirs. These long-lived HIV-1 reservoirs evade the immune response and persist for long periods, even in the presence of successful HAART. In fact, they represent a major obstacle for the eradication of HIV-1 in HAART-treated patients [8,9].

The capacity of infected macrophages to produce HIV-1 can be modulated in several ways. Activation of TLR3, 4, 7 and 8 arrests HIV-1 infection after virus entry, but before reverse transcription [10]. In contrast, polybacterial challenge enhances HIV-1 reactivation in latently infected macrophages and DCs [11]. Cytokine-induced polarization of macrophages into classical activated macrophages results in a reduced capacity to support a productive infection due to the inhibition of an early preintegration step in the viral life cycle [12]. Polarization into alternatively activated macrophages also results in HIV-1 production decrease. However, in this case, inhibition occurs at a late postintegration level in the viral life cycle [12]. On the other hand, contradictory results have been reported on the ability of other agents such as *M. tuberculosis* or LPS to modulate the rate of HIV-1 production by infected macrophages [13–17].

Candida albicans (CA) is a polymorphic fungus and a commensal microorganism in the healthy subject that colonizes the human gastrointestinal tract, the oral cavity and the vagina. However, in the immunocompromised host, CA becomes an aggressive pathogen. In fact, oropharyngeal candidiasis affects up to 50% of untreated HIV-1+ subjects and 90% of AIDS patients [18,19]. Interestingly, HIV-1 directly interacts with CA and this interaction appears to enhance fungal virulence [20,21]. Moreover, HIV-1 has shown to inhibit phagocytosis of CA mediated by macrophages [22]. In this study, we analyzed the ability of CA to modulate the course of HIV-1 infection in monocyte-derived macrophages. We found that CA abrogates the early production of HIV-1 by infected macrophages favoring the establishment of a silent infection.

## Materials and Methods

### Reagents

Trypsin, zymosan A from *Saccharomyces cerevisiae*, and recombinant human granulocyte-macrophage colony-stimulating factor (GM-CSF) were from Sigma-Aldrich (St. Louis, MO, USA). Recombinant human interleukin-4 (IL-4) was from Preprotech (Rocky Hill, NJ, USA) or R&D Systems (Minneapolis, MN, USA). Ficoll-Hypaque was from Amersham Pharmacia Biotech (Piscataway, NJ, USA). RPMI 1640 medium supplemented with 10% heat-inactivated fetal calf serum (FCS), 50 U of penicillin/ml and 50 µg of streptomycin/ml (Life Technologies, Grand Island, NY, USA) was used as culture medium. This medium was supplemented with anfostat (1µg/ml) (Life Technologies) in those experiments performed with CA.

### Preparation of macrophages and dendritic cells

Human peripheral blood mononuclear cells (PBMCs) were obtained from healthy volunteers after provision of written informed consent, and in accordance with the principles of the Declaration of Helsinki, through protocols approved by the Institutional Board from the National Academy of Medicine (Buenos Aires, Argentina). All the research was conducted in Argentina. PBMCs were isolated from blood of healthy donors by density gradient centrifugation on Ficoll-Hypaque. CD14^+^ cells were obtained using CD14 microbeads (Miltenyi Biotec, Auburn, CA, USA). To obtain DCs, monocytes were cultured for 5 days with 20 ng/ml IL-4 and 10 ng/ml GM-CSF [2,3]. To obtain macrophages, monocytes were cultured with 20 ng/ml GM-CSF for 5 days [2,4].

### Cell lines and virus

The R5 tropic virus HIV-1_BaL_ [2,5], the X4 tropic virus HIV-1_IIIB_ [2,5], and the T cell line Jurkat [2,6] were obtained from the AIDS Research and Reference Reagent Program, Division of AIDS, National Institute of Allergy and Infectious Diseases, National Institutes of Health. The HIV-1_BaL_ strain was grown on monocyte-derived macrophages and the HIV-1_IIIB_ strain was obtained from H9HTLV_IIIB_ supernatants, as described [2,7]. Viruses were concentrated by ultracentrifugation at 28,000 rpm for 90 min at 4°C (L2-65B ultracentrifuge; Beckman Coulter, Brea, CA, USA), and the virus pellet was suspended in RPMI 1640 medium. p24 antigen levels were determined by ELISA (bioMérieux, Vironostika, Buenos Aires, Argentina), and the virus input into assays was a function of p24 antigen concentration.

### Flow cytometry

Fluorescein isothiocyanate- phycoerythrin-, or allophycocyanin- conjugated mAbs directed to CD1a, CD14, CD4, DC-SIGN, CD86, CD83, HLA-DR, CCR7, and CCR5 were from BD Pharmingen (San Diego, CA, USA). Anti-CD317 (BST-2) was from eBioscience (San Diego, CA, USA). A fluorescein isothiocyanate (KC57-FITC) conjugated mAb directed to HIV-1 proteins 55, 39, 33, and 24kD of core antigen was from Beckman Coulter (Fullerton, CA, USA). The analysis was performed using a FACS flow cytometer and the CellQuest software (BD Biosciences, San Jose, CA, USA).

### Quantification of cellular apoptosis by annexin-V binding and flow cytometry

It was performed using an apoptosis detection kit (Immunotech, Marseille, France). In brief, cells were labeled with annexin-V-FITC for 20 min at 4^°^C and with propidium iodide immediately before evaluation by flow cytometry [2,8].

### HIV-1 binding assays

They were performed as previously described [2,7]. Briefly, CA (1x10^6^ to 1x10^7^) or zymosan (100 µg) suspended in 0.5 ml of culture medium were incubated with HIV-1 (50 ng p24/ml) for 60 min at 37°C, washed thoroughly, pelleted, lysed, and assayed for p24 antigen by ELISA.

### HIV-1 infection assays

They were performed as previously described [2,7]. Adherent macrophages, DCs, or Jurkat cells (5x10^5^/0.5ml) were cultured in 24 flat-bottom plates with the R5 tropic virus HIV-1_BaL_ or the X4 tropic virus HIV-1_IIIB_ (50 ng p24/ml) for 2 h at 37^°^C, in the presence or absence of CA (ATCC10261) or zymosan. Cells were then washed three times with PBS and cultured for different periods. Infection was analyzed by measuring the concentration of p24 antigen in cell supernatants by ELISA or by studying the expression of intracellular HIV-1 core proteins by flow cytometry. To neutralize the activity of type I IFNs, a specific blocking antibody directed to the common receptor IFN-R was used (clone MMHAR-2, PBL interferon Source, NJ, USA).

### Transmission of HIV-1 from macrophages to activated mononuclear cells

PBMCs were activated by IL-2 (10 U/ml; R&D Systems) and PHA (10µg/ml) for 2 days. Macrophages (5x10^5^/0.5 ml) were cultured in 24 flat-bottom plats with the R5 tropic virus HIV-1_BaL_ (50 ng p24/ml) for 2 h at 37^°^C, in the absence or presence of CA (macrophage: CA ratio of 1:10). Macrophages were then washed thoroughly and cultured in medium supplemented with 10 ng/ml of GM-CSF. Transmission assays were performed as previously described [2,7] by incubating 5x10^5^ HIV-1-treated macrophages with 2.5x10^5^ activated PBMCs in a final volume of 0.5 ml in 24 flat-bottom plats. Supernatants harvested at 4 and 8 days of culture were assayed for p24 antigen by ELISA.

### Confocal microscopy

Macrophages (5x10^5^ cells/0.5ml) were cultured with or without FITC labeled-CA for 18 h (macrophage: CA ratio of 1:10). Macrophages were then harvested and plated on poly-L-lysine-coated glass coverslips (12 mm) during 20 min at RT. Then, cells were washed and fixed in 4% paraformaldehyde (10 min on ice) and washed twice with 0.1 mM glycine in PBS. Subsequently, cells were incubated with a mouse mAb directed to CD4 for 1 h, and revealed with DyLight549-conjugated anti-mouse mAb for 45 min. Coverslips were mounted on glass slides using Fluoromount G. Immunofluorescence and images were acquired with a FluoView FV1000 confocal microscope (Olympus, Tokyo, Japan) using a Plapon 60-1.42 NA oil immersion objective. Images were analyzed using the Olympus FV10-ASW software.

### HIV-1 DNA detection

Total macrophage DNA was isolated 18 h post-infection using the DNeasy Blood and Tissue Kit (QIAGEN, Alameda, CA) according to the manufacturer’s instructions. Two different HIV-1 genomic regions were targeted for PCR amplification: the *gag* region (132 bp) and the *env* region (322 bp). Nested PCR was then performed with Taq DNA polymerase and buffers (Invitrogen, Carlsbad, CA, USA), under the conditions recommended by the manufacturer. Primers used for first-round amplification were (**Gag 1** 5’-GAA GGC TTT CAG CCC AGA AG-3’; **Gag 2** 5’-TCT CCT ACT GGG ATA GGT GG-3’; **Env 1** 5’-CAC AGT ACA ATG TAC ACA TG-3’; **Env 2** 5’-ACA GTA GAA AAA TTC CCC TC-3’). β-Actin was amplified as a control (**Act 1** 5’-GGA CCT GAC TGA CTA CCT CAT GAA-3’; **Act 2** 5’-GAT CCA CAT CTG CTG GAA GGT GGG AG-3’). The following amplification conditions were used: 5 min at 95°C, followed by 25 cycles of 30s at 95°C, 30s at 60°C, 30s at 75°C, and a final extension step at 72°C for 5 min. Primers used for second-round amplification were (**Gag 3** 5’-ACC ATC AAT GAG GAA GCT GC-3’; **Gag 4** 5’-TAT TTG TTC CTG AAG GGT AC-3’; **Env 3** 5’-AAA TGG CAG TCT AGC AGA AG-3’; **Env 4** 5’-ACA ATT TCT GGG TCC CCT CC-3’), following the same conditions to those used in the first round.

### RNA extraction, cDNA synthesis and quantitative Real-Time PCR

Total RNA was extracted from 2x10^6^ macrophages using TRIzol® Invitrogen, according to the manufacturer’s instructions. It was transcribed to cDNA using random primers and M-MLV RT (Invitrogen). Specific primer pairs for each gene (Invitrogen) were designed: **CCL3** (GI: 121582465) (**fw** 5’-AGTTCTCTGCATCACTTGCTG-3’, **rev** 5’- CGGCTTCGCTTGGTTAGGAA-3’); **CCL4** (GI: 59799728) (**fw** 5’-CTGTGCTGATCCCAGTGAATC-3', **rev** 5’-TCAGTTCAGTTCCAGGTCATACA-3’); **CCL5** (GI: 22538813) (**fw** 5’-ATCCTCATTGCTACTGCCCTC-3’, **rev** 5’-GCCACTGGTGTAGAAATACTCC-3’); **APOBEC3F** (GI: 109451045) (**fw** 5’-TACGCAAAGCCTATGGTCGG-3’, **rev** 5’-GCTCCAAGATGTGTACCAGC-3’); **APOBEC3G** (GI: 109451183) (**fw** 5’-GCTGTGCTTCCTGGACGTGA-3’, **rev** 5’-GGTGGTCCACAAAGGTGTCCC-3’); **GAPDH** (GI: 354515022) (**fw** 5’-GAGTCAACGGATTTGGTCGT-3’, **rev** 5’-TTGATTTTGGAGGGATCTCG-3’). Gene expression analysis was performed on a 7500 Real Time PCR System (Applied Biosystems, Foster, CA, USA) using SYBR®Green PCR Master Mix for detection of PCR product (Applied Biosystems). All reactions were performed in duplicate with appropriate non-template controls. Finally, a melting curve analysis was performed to verify the specificity of the amplicon.

### Statistical analysis

All statistical comparisons were performed using one-way analysis of variance with Dunnett post-test. P values <0.05 were considered statistically significant.

## Results

### Candida albicans inhibits the replication of HIV-1 in macrophages and dendritic cells

**Figure 1:**
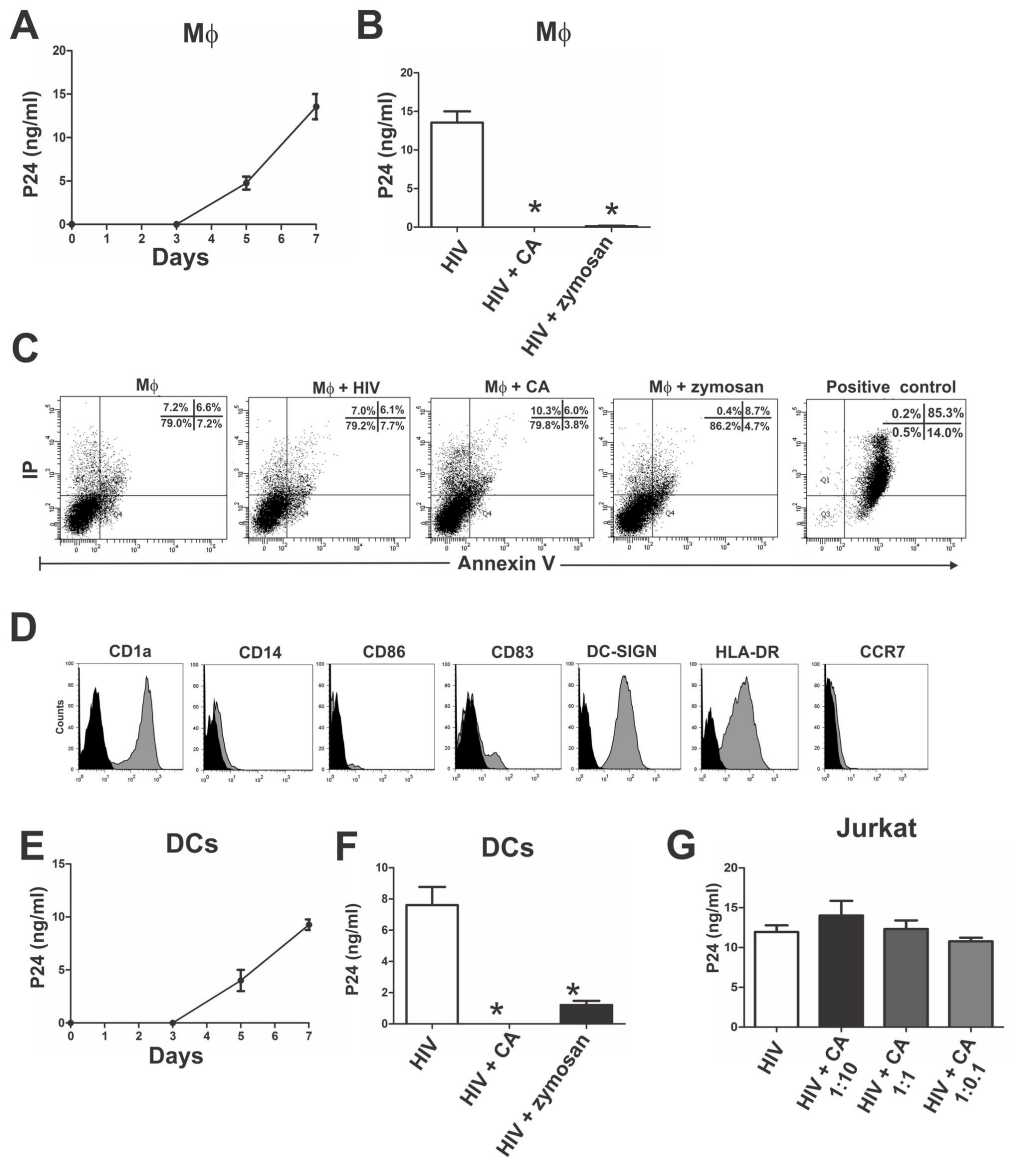
*Candida albicans* abrogates the production of virus particles by HIV-1-challenged macrophages. (**A**) Macrophages (5x10^5^/0.5 ml) were cultured in 24 flat-bottom plats with the R5 tropic virus HIV-1_BaL_ (50 ng p24/ml) for 2 h. Then, cells were washed and the production of HIV-1 was evaluated at days 0, 3, 5 and 7 post-infection by measuring the amount of p24 antigen in cell supernatants (n=4). (**B**) Macrophages were cultured with HIV-1_BaL_ (50 ng p24/ml) for 2 h in the absence or presence of CA (macrophage: CA ratio of 1:10) or zymosan (100 µg/0.5 ml). Then, cells were washed and the production of HIV-1 was evaluated after 7 days (n=9). (**C**) Macrophages were cultured for 7 days in the absence or presence of HIV-1_BaL_ (50 ng p24/ml), CA (macrophage: CA ratio of 1:10) or zymosan (100 µg/0.5 ml). Then, the viability of macrophages was analyzed by flow cytometry using Annexin V and propidium iodide. Positive control of necrosis represents macrophages cultured for 3 days in protein-free medium (n=3). (**D**) Histograms illustrate the phenotype of DCs used in our experiments (n=5). (**E**) DCs (5x10^5^/0.5 ml) were cultured with HIV-1_BaL_ (50 ng p24/ml) for 2 h. Then, cells were washed and the production of HIV-1 was evaluated at days 0, 3, 5 and 7 post-infection (n=4). (**F**) DCs were cultured with HIV-1_BaL_ (50 ng p24/ml) for 2 h in the absence or presence of CA (macrophage: CA ratio of 1:10) or zymosan (100 µg/0.5 ml). Then, cells were washed and the production of HIV-1 was evaluated after 7 days of culture (n=5). (**G**) The T cell line Jurkat (5x10^5^/0.5 ml) was incubated with the X4 tropic virus HIV-1_IIIB_ (50 ng p24/ml) and CA (Jurkat: CA ratios of 1:10, 1:1, and 1:0.1) for 2 h. Cells were then washed and HIV infection was revealed after 7 days (n=3). In all cases, results are expressed as the arithmetic means ± SEM of n experiments or are illustrated as representative dot-plot or histograms. *p<0.05 vs HIV.

In a first set of experiments we analyzed the kinetics of HIV-1 infection in monocyte-derived macrophages. Macrophages were cultured with the R5 tropic virus HIV-1_BaL_ for 2 h. Then, cells were washed and the infection of macrophages was evaluated at days 0, 3, 5 and 7 by measuring the amount of p24 antigen in cell supernatants by ELISA. Significant amounts of p24 antigen were detected after 5 days of infection (Figure **1A**). We then analyzed whether CA was able to modulate the infection of macrophages by HIV-1. Macrophages were cultured with HIV-1_BaL_ in the absence or presence of CA at a macrophage: CA ratio of 1:10. After 2 h of culture, cells were washed and HIV-1 infection was evaluated after 7 days of culture. Figure **1B** shows that CA abrogated the production of HIV-1 by macrophages. A similar inhibitory effect was observed using zymosan, a product prepared from the cell wall of the fungus *Saccharomyces cerevisiae*. To rule out the possibility that the decreased production of HIV-1 could be related to a deleterious effect induced by CA, cellular viability was evaluated by annexin-V binding and flow cytometry. Figure **1C** shows that neither CA nor zymosan diminished macrophage viability.

We then analyzed whether CA was also able to inhibit HIV-1 replication in monocyte-derived dendritic cells (DCs). The phenotype of DCs is illustrated in Figure **1D**. As expected, DCs were CD1a^+^, CD14^-^, DC-SIGN^+^, and showed predominantly an immature phenotype illustrated by the low expression of CD86 and the absence of CCR7. However, a fraction of DCs (5-20%) were positive for the expression of CD83 indicating the presence of a population of mature DCs. The kinetics of HIV-1 infection revealed that significant amounts of p24 were detected in cell supernatants after 5 days of infection (Figure **1E**). Similarly to macrophages, the production of HIV-1 by DCs was markedly reduced by either CA or zymosan (Figure **1F**). In contrast with the findings observed in macrophages and DCs, CA did not exert any inhibitory effect on the infection of the Jurkat T cell line by the X4 tropic virus HIV-1_IIIB_ (Figure **1G**).

**Figure 2:**
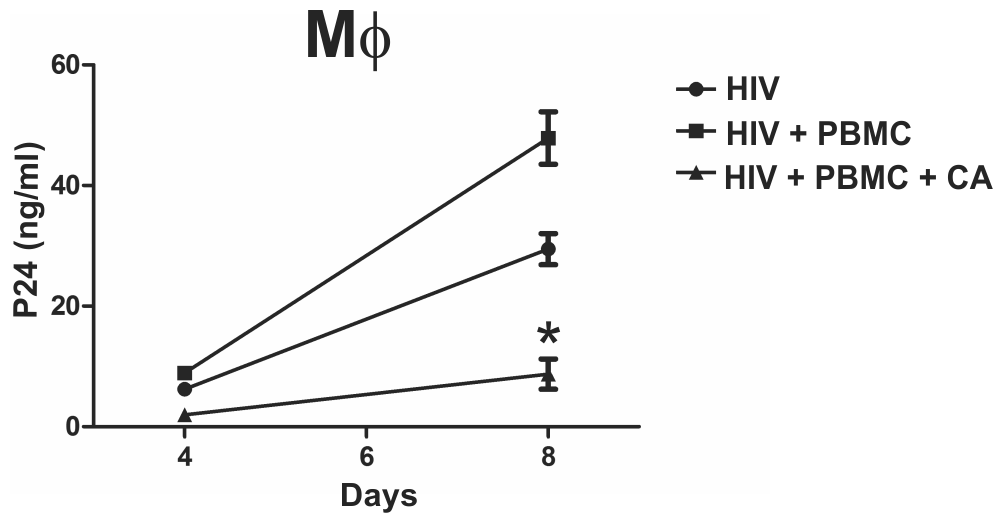
*Candida albicans* inhibits transmission of HIV-1 from macrophages to activated peripheral blood mononuclear cells. Macrophages (5x10^5^/0.5 ml) were incubated with the R5 tropic virus HIV-1_BaL_ (50 ng p24/ml) for 2 h in the absence or presence of CA (macrophages: CA ratio of 1:10). Cells were then washed and cultured with activated PBMCs (2.5 x10^5^). Infection was revealed after 4 and 8 days of culture by measuring the amount of p24 antigen in cell supernatants by ELISA. Results are expressed as the arithmetic means ± SEM of 4 experiments. * p<0.05 vs HIV + PBMC.

We then evaluated whether CA also impaired the transmission of the virus from macrophages to activated peripheral blood mononuclear cells (PBMCs). In these experiments macrophages were cultured for 2 h with the R5 tropic virus HIV-1_BaL_ in the absence or presence of CA (macrophage: CA ratio of 1:10). Cells were then washed and cultured with activated PBMCs using a macrophage: PBMC ratio of 2:1. The production of HIV-1 was evaluated at days 4 and 8 post-infection. Results in Figure **2** show that CA markedly inhibited the production of HIV-1 in cocultures of macrophages and activated PBMCs.

### Analysis of the mechanisms through which Candida albicans inhibit HIV-1 replication in macrophages

**Figure 3:**
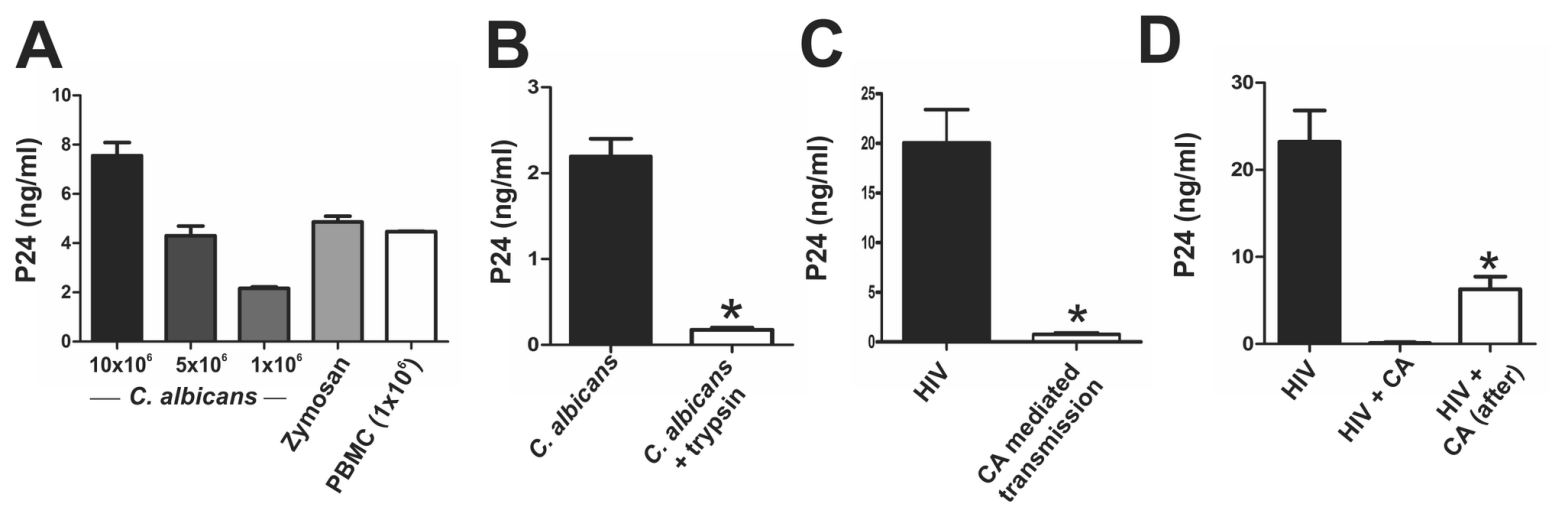
*Candida albicans* sequesters HIV-1 particles. (**A**) CA (1x10^6^-1x10^7^/0.5 ml), zymosan (100 µg/0.5 ml), or activated PBMCs (1x10^6^/0.5 ml) were incubated with HIV-1_BaL_ (50 ng p24/ml) for 2 h, washed thoroughly, lysed, and assayed for p24 antigen by ELISA. A representative experiment (n=4) is shown. (**B**) CA (1x10^6^/0.5 ml) was incubated with HIV-1_BaL_ (50 ng p24/ml) for 2 h, washed thoroughly, treated with trypsin (1000 U/ml, 15 min at 37°), lysed and assayed for p24 antigen. Bars represent the arithmetic means ± SEM of four experiments. * p<0.05 vs *C. albicans*. (**C**) CA (5x10^6^/0.5 ml) was incubated with HIV-1_BaL_ (50 ng p24/ml) for 2 h and washed thoroughly. Macrophages (5x10^5^/0.5 ml) were cultured for 7 days with HIV-treated CA and the levels of p24 antigen in cell supernatants was assayed by ELISA. Bars represent the arithmetic means ± SEM of four experiments. * p<0.05 vs macrophages infected by HIV-1. (**D**) Macrophages (5x10^5^/0.5 ml) were incubated with HIV-1_BaL_ (50 ng p24/ml) for 2 h in the presence (HIV + CA) or absence (HIV + CA after) of CA, and washed three times with PBS. Cells were then cultured for an additional period of 2 h in the absence (HIV + CA) or presence (HIV + CA after) of CA. Cells were washed again and HIV-1 infection was evaluated after 7 days. The macrophage: CA ratio used in these experiments was 1:10. Bars represent the arithmetic means ± SEM of 6 experiments. * p<0.05 vs HIV + CA.

We speculated that the inhibition of HIV-1 infection induced by CA might involve the sequestering of HIV-1 particles by CA. Supporting this possibility, previous studies have shown that CA binds HIV-1 through a mechanism depending on the interaction between the C3-like regions of the HIV-1 transmembrane protein gp41 and C3 binding moieties on CA [20,21]. Consistent with these observations, we found that both CA and zymosan efficiently bind HIV-1 (Figure **3A**). In fact, 5x10^6^ CA bind a similar amount of HIV-1 than 1x10^6^ PBMCs (Figure **3A**). The attached virus remained at the yeast surface (it was removed by trypsin) (Figure **3B**) and was completely unable to start a productive infection in macrophages (Figure **3C**). To further analyze whether the sequestering of HIV-1 by CA could account for the inhibition of HIV-1 infection, a new set of experiments was performed under conditions directed to minimize the interaction between CA and HIV-1. To this aim, macrophages were incubated with HIV-1 for 2 h. Cells were washed and CA was then added using a macrophage: CA ratio of 1:10. Infection of macrophages was revealed at day 7 post-infection. Results in Figure **3D** show that, even under these experimental conditions, CA inhibited the production of HIV-1. However, the inhibitory effect was significantly lower compared with the inhibition of HIV-1 production observed when macrophages were cultured together with both CA and HIV-1 (Figure **3D**). This suggests that sequestering of HIV-1 particles by CA might contribute to the inhibition of HIV-1 infection. Also supporting this view, we found that when HIV-1 stocks were pre-incubated with CA and the infectivity of these stocks in macrophages were measured after depletion of CA by centrifugation, a significant inhibition was observed (data not shown).

**Figure 4:**
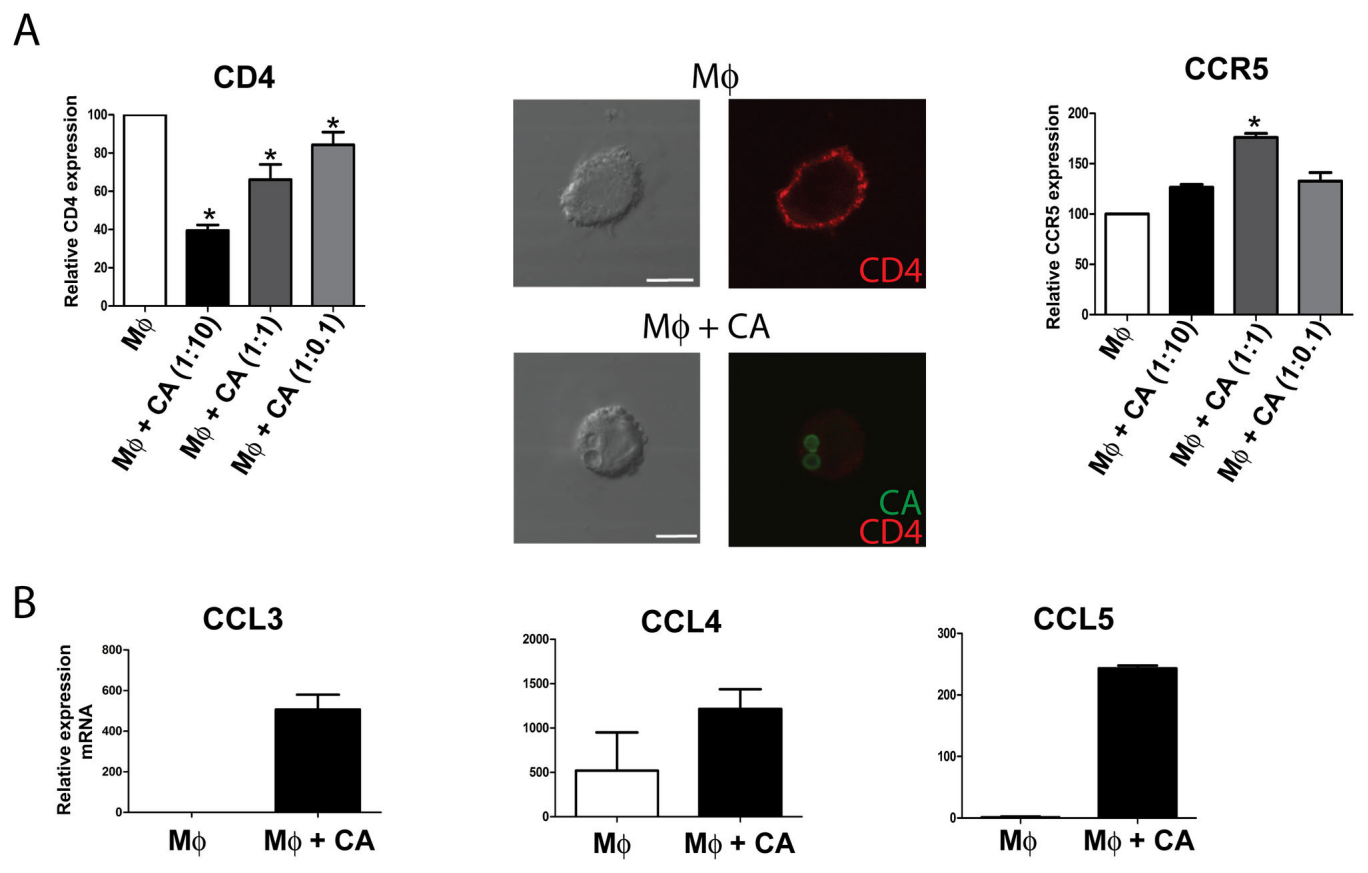
*Candida albicans* down-regulates CD4 expression and stimulates the production of the CCR5-ligand chemokines CCL3, CCL4, and CCL5 in macrophages. Macrophages (5x10^5^/0.5 ml) were cultured for 2 h in the absence or presence of CA using a macrophage: CA ratio of 1:10, unless otherwise stated. Cells were then washed and cultured for an additional period of 18 h. Then, the expression of CD4 and CCR5 was analyzed by flow cytometry or confocal miscroscopy (**A**) and the production of the chemokines CCL3, CCL4 and CCL5 was evaluated in cell supernatants by quantitative PCR (relativized to the expression of GAPDH mRNA) (**B**). In (**A**), graph bars show the relative mean fluorescence intensity (MFI) of CD4 or CCR5. The MFI of untreated macrophages is assigned the value of 100. Results are expressed as the arithmetic means ± SEM of 4-5 experiments. Confocal images of a representative experiment (n=4) are also shown. Bars represent 10 µm. In (**B**), representative experiments are shown (n=4).

We then analyzed whether the ability of CA to suppress HIV-1 replication could also be related to changes in the phenotype of macrophages. We first looked at the expression of CD4 and CCR5. Macrophages were incubated with CA for 2 h at different macrophage: CA ratios, washed and cultured for 18 h. Then, the expression of CD4 and CCR5 was analyzed by flow cytometry or confocal microscopy. Figure **4A** shows that CA markedly inhibited the expression of CD4 while slightly increased the expression of CCR5. Considering that the CC chemokines CCL3/MIP-1α, CCL4/MIP-1β, and CCL5/RANTES block the entry of R5 tropic virus into host cells [2,9], we also analyzed the levels of mRNA for these chemokines. Figure **4B** shows that activation of macrophages by CA stimulated the production of CCL3, CCL4, and CCL5, as revealed by quantitative PCR.

**Figure 5:**
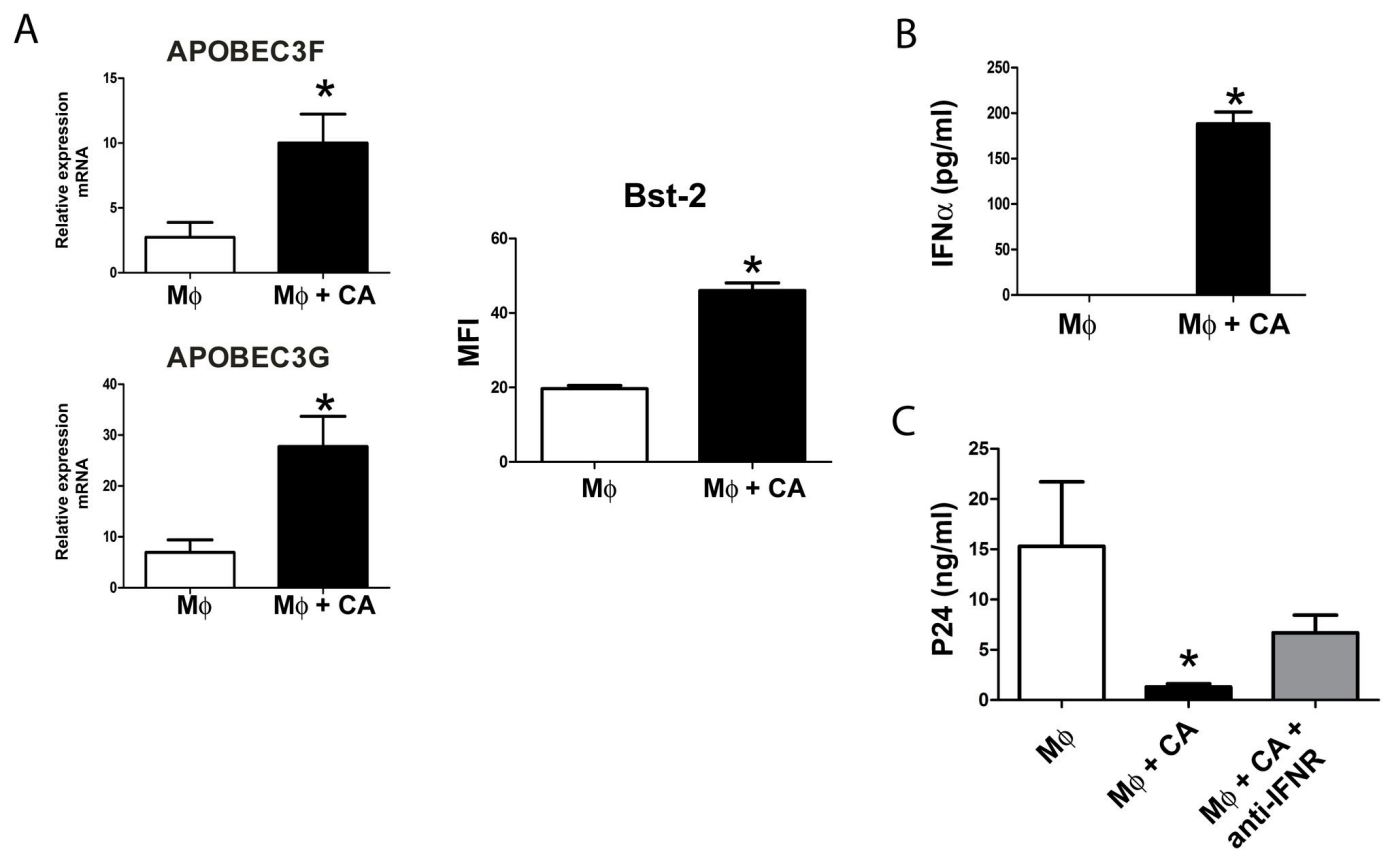
*Candida albicans* stimulates the production of the retroviral host-restriction factors APOBEC3G, APOBEC3F, tetherin, and interferon-α. (**A** and **B**) Macrophages (5x10^5^/0.5 ml) were cultured for 2 h in the absence or presence of CA using a macrophage: CA ratio of 1:10. Cells were then washed and cultured for an additional period of 18 h. Then, the presence of APOBEC3F and APOBEC3G was evaluated by quantitative PCR (relativized to the expression of GAPDH mRNA), the expression of tetherin (Bst-2) analyzed by flow cytometry, and the amount of IFN-α quantified in cell supernatants by ELISA. (**C**) Macrophages (5x10^5^/0.5 ml) were cultured with HIV-1_BaL_ (50 ng p24/ml) in the absence or presence of CA (macrophage: CA ratio of 1:10). Then, cells were washed and incubated with or without saturating concentrations of a blocking monoclonal antibody directed to the receptor for type I IFNs. The production of HIV-1 was evaluated at day 7 post-infection. In (**A**), representative experiments are shown (4,5). Bars represent the arithmetic means ± SEM of 5 experiments. (A and B) *p<0.05 vs M. (**C**) *p<0.05 vs M or M+CA+anti-IFNR.

The inhibition of HIV-1 replication in macrophages induced by CA could also be related to an increased expression of host restriction factors able to block retroviral replication. APOBEC3G and APOBEC3F are cytidine deaminases that introduce G to A substitutions in the HIV-1 genome inhibiting viral replication [30]. On the other hand, tetherin (Bst-2) impairs the release of virions by “tethering” mature virions to the cell membrane [31]. We then analyzed whether CA was able to stimulate the expression of these retroviral restriction factors in macrophages. In these experiments, cells were incubated for 2 h at a macrophage: CA ratio of 1:10, washed and cultured for 18 h. Then, the presence of mRNA for APOBEC3F and APOBEC3G was evaluated by quantitative PCR while the expression of tetherin was analyzed by flow cytometry. Figure **5A** shows that CA stimulated the expression of APOBEC3F, APOBEC3G and tetherin. Considering that type I IFNs represent the most potent inducer of these restriction factors, we looked at the production of interferon-α. Figure **5B** shows that CA effectively stimulated the production of interferon-α by macrophages. Interestingly, the addition of saturating concentrations of a blocking monoclonal antibody directed to the receptor for type I IFNs partially prevented the inhibition of HIV-1 infection in macrophages induced by CA (Figure **5C**), suggesting the participation of type I IFNs in the anti-viral effect induced by CA.

### *Candida albicans* promotes and sustains a latent-like HIV-1 infection in macrophages

**Figure 6:**
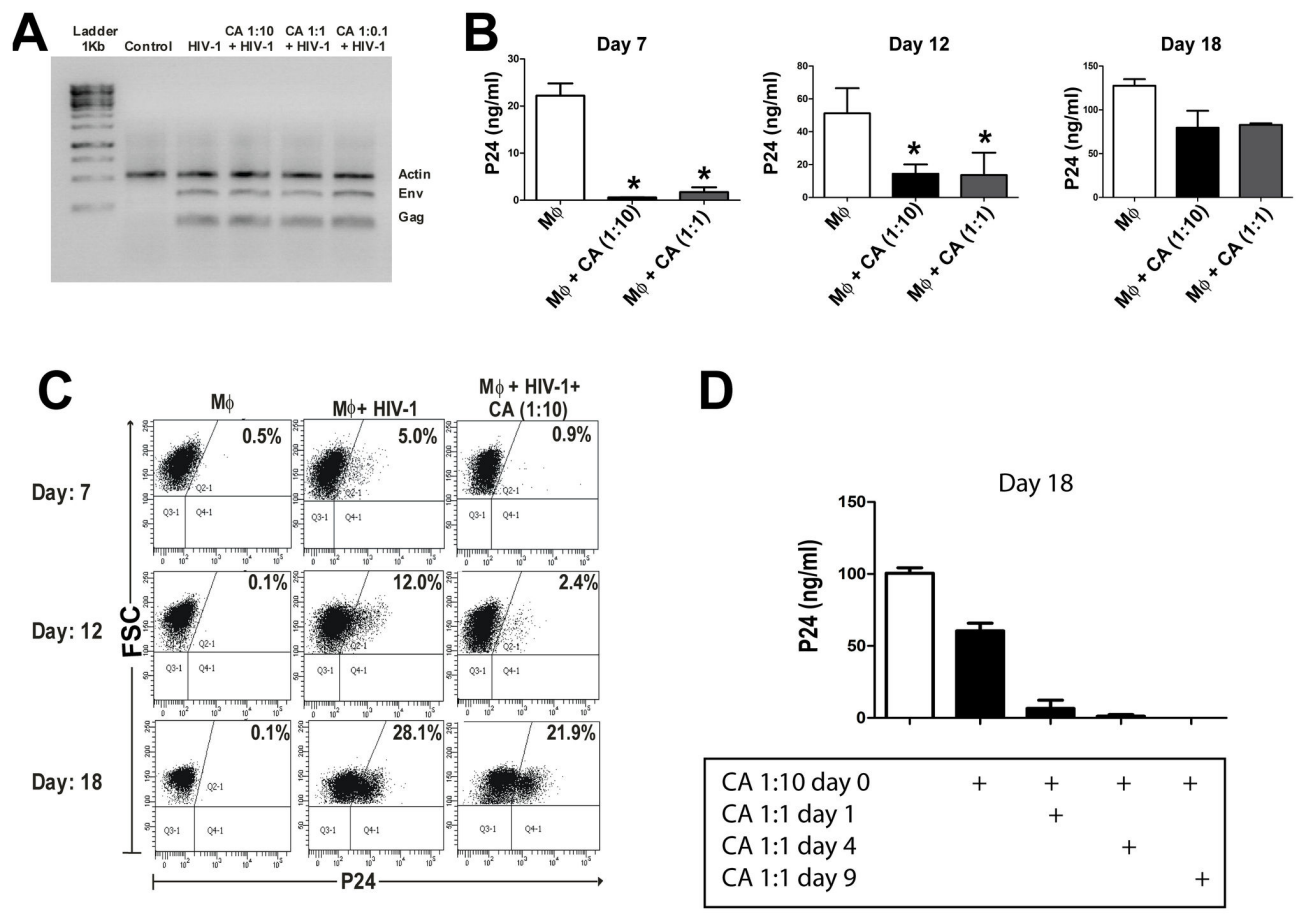
*Candida albicans* promotes and sustains a latent-like HIV-1 infection in macrophages. (**A**) Macrophages (5x10^5^/0.5 ml) were cultured with HIV-1_BaL_ (50 ng p24/ml) for 2 h at different macrophage: CA ratios. Then, cells were washed thoroughly and cultured for an additional period of 18 h. PCR amplification of HIV-1 DNA (env, and gag) was performed in cell lysates and β-actin was evaluated as a control. A representative experiment (n=3) is shown. (**B** and **C**) Macrophages (5x10^5^/0.5 ml) were cultured for 2 h with HIV-1_BaL_ (50 ng p24/ml) in the absence or presence of CA (macrophage: CA ratios of 1:10 and 1:1). Then, cells were washed and the production of HIV-1 was evaluated at 7, 12, and 18 days post-infection in cell supernatants by p24 ELISA (**B**) or by intracellular staining of p24 antigen and flow cytometry (**C**). (**B**) Graph bars represent the arithmetic means ± SEM of 4 experiments carried out in duplicate. * p<0.05 vs Mϕ. (**C**) Dot plots from a representative experiment (n=3) are shown. (**D**) Macrophages (5x10^5^/0.5 ml) were cultured with HIV-1_BaL_ (50 ng p24/ml) for 2 h in the absence or presence of CA (macrophage: CA ratio of 1:10). Then, cells were washed thoroughly and incubated in culture medium. At days 1, 4, or 9 after the initial challenge by HIV-1, cell cultures were supplemented with additional amounts of CA (macrophage: CA ratio of 1:1), and the production of HIV-1 was evaluated in all cases at day 18 post-infection in cell supernatants by ELISA. Graph bars represent the arithmetic means ± SEM of 5 experiments. * p<0.05 vs Mϕ cultured without CA (white bar) and ** p<0.05 vs Mϕ + CA (day 0).

Results in Figure **6A** show that HIV-1 DNA was similarly detected in macrophages challenged by HIV-1 either in the absence or presence of CA, suggesting that the inhibitory effect of CA on HIV-1 replication is related to the inhibition of a post-entry step of the viral life cycle. We then asked whether macrophages would be able to overcome this form of latency. To answer this question, we next performed kinetic studies in which the production of viral particles was analyzed at days 7, 12 and 18 post-exposure to HIV-1. In these experiments, macrophages were cultured with HIV-1_Bal_ in the absence or presence of CA (macrophage: CA ratios of 1:10 and 1:1). Cells were washed and infection was revealed at days 7, 12, and 18. The results obtained are shown in Figure **6B and C**. *Candida albicans* markedly inhibited the production of HIV-1 when evaluated at either 7 or 12 days post-exposure. However, this inhibitory effect was largely overcome when infection was evaluated 18 days post-infection. Interestingly, as shown in Figure **6D**, the delayed reactivation of HIV-1 infection could be silenced by longer periods when CA was added periodically to HIV-1-challenged macrophages.

## Discussion

Whereas HIV-1 can infect a variety of cell types, CD4^+^ T cells, macrophages and DCs represent the three main targets of HIV-1 infection [1,2]. Although the virus follows a similar life cycle in CD4^+^ T lymphocytes and macrophages, the infection of both cell types shows major differences. One of the most striking differences consists in the high resistance of macrophages to the cytopathic effects of HIV-1. CD4^+^ T cells rapidly die by apoptosis upon acute infection showing a half-life of a few days [32,33]. In contrast, infected macrophages survive for months after acute infection, and as a result they can accumulate and produce high levels of virus for long periods of time [3–5].

The introduction of the HAART in 1996 unveiled the presence of latent and long-lived viral reservoirs which constitute a major obstacle for HIV-1 eradication. Both, CD4^+^ resting T cells (naive and memory) and macrophages seem to be the most important reservoirs for HIV-1, and the presence of latent proviral DNA has been clearly demonstrated in these cellular populations [8,9,3,4]. A large number of studies have been performed to analyze the mechanisms responsible for the induction and maintenance of HIV-1 latency in CD4^+^ T cells, while less attention has been paid to the analysis of latency in macrophages. However, for both cell types two forms of latency have been described: pre-integration latency and post-integration latency [5,8,9].

A variety of stimuli have shown to modulate *in vitro* the course of HIV-1 infection in macrophages. IFN-α potently inhibits HIV-1 replication in macrophages while IL-1β and IL-6 stimulate virus replication [35,3,6]. IL-4 and IL-13 enhance virus replication in monocytes but suppress HIV-1 replication in macrophages [3,7]. Polarization of macrophages into a M1 profile induced by TNF-α plus IFN-γ leads to a decreased capacity to support HIV-1 replication [12,38]. Contradictory results have been published in regard to the ability of *M. tuberculosis* to modulate the course of HIV-1 infection in macrophages [13,14], while *M. avium* and *M* *. xenopi* have shown to enhance HIV-1 replication [39,40]. Gram-negative polybacterial challenge enhances HIV-1 replication in macrophage and DCs [11] while phagocytosis of apoptotic cells stimulates HIV-1 replication in mononuclear phagocytes [41].

In this study we show that the fungus CA almost completely abrogates the early production of viral particles by macrophages and DCs challenged by HIV-1. Not only the production of HIV-1 but also the ability of macrophages to transmit the virus to activated mononuclear cells were abolished by CA. Our results suggest that CA suppresses HIV-1 production in macrophages by a number of mechanisms. First, it efficiently attaches HIV-1 particles. In contrast with previous observations made in other non-permissive cells such as epithelial cells [42], spermatozoa [27] and erythrocytes [43] indicating that attached virions are efficiently transmitted to macrophages and DCs, promoting the spreading of HIV-1 infection, we found that CA-attached HIV-1 was completely unable to start a productive infection in macrophages, perhaps reflecting the ability of CA to drive the virus into a degradation pathway inside macrophages. Moreover, we speculate that CA might also inhibit the infection of macrophages and DCs not only by sequestering HIV-1 particles but also by competing with HIV-1 for binding sites on the cell surface, a possibility that should be tested experimentally. Secondly, CA markedly reduced the expression of CD4 while enhanced the production of the CCR5 chemokine ligands CCL3/MIP-1α, CCL4/MIP-1β, and CCL5/RANTES. Thirdly, CA stimulated the production of interferon-α and the restriction factors APOBEC3G, APOBEC3F, and tetherin. Moreover, supporting a role for type I IFNs we found that blocking of the receptor for type I IFNs partially, but significantly prevented the inhibitory effect of CA on the production of HIV-1 by infected macrophages.

Two previous reports analyzed the effect of CA on HIV-1 infection in DCs [44,4,5]. Both studies showed that CA partially restricts HIV-1 amplification in DCs while increases DC to T-cell transmission of HIV-1. Contrasting with these observations, our results in macrophages showed that CA abrogates not only the replication of HIV-1 in macrophages but also their ability to transmit the virus to activated PBMCs. This suggests that CA exerts opposite effects on the ability of macrophages and DCs to transmit HIV-1 infection to CD4^+^ T cells.

We also found that the inhibitory effect induced by CA on the production of HIV-1 by macrophages was overcome when infection was evaluated 18 days after virus inoculation. Interestingly, this delayed reactivation of HIV-1 infection in macrophages could be silenced by longer periods when CA was added periodically to cell cultures thus suggesting that CA might promote and sustain a latent-like HIV-1 infection in macrophages.

*Candida albicans* normally colonizes the portals of HIV-1 entry, such as the vagina and the rectum. Moreover, mucocutaneous candidiasis is one of the most common manifestations of HIV-1 infection [18,19]. Since CA can infect, survive and persist in macrophages [46–44,8], it is reasonable to speculate that coinfection of macrophages by HIV-1 and CA might occur in infected patients in the periphery, at the portals of HIV-1 entry. In this scenario, suppression of HIV-1 replication in macrophages induced by CA could induce opposite effects on the course of HIV-1 infection. It might prevent the spreading of HIV-1 by macrophages. However, the induction of a silent infection in the macrophage might help HIV-1 to evade the immune response and to promote resistance to antiretroviral therapy. Further studies are needed to establish whether coinfection of macrophages by both HIV-1 and CA occurs in coinfected patients as well as the influence of CA on HIV-1 infection of macrophages in vivo.
